# Effects of a Tele-Prehabilitation Program with Indirect Electrostimulation Compared to Home-Based Exercise in Patients Eligible for Lower Limb Arthroplasty: A Randomized Controlled Trial

**DOI:** 10.3390/jcm14041356

**Published:** 2025-02-18

**Authors:** Pamela Patanè, Vittoria Carnevale Pellino, Massimiliano Febbi, Caterina Cavallo, Fabrizio Gervasoni, Alessandro Gatti, Emanuele Caldarella, Francesca de Caro, Matteo Vandoni, Federica Manzoni, Luca Marin

**Affiliations:** 1 Laboratory for Rehabilitation Medicine and Sport (LARMS), 00133 Rome, Italy; pamela.patane95@gmail.com (P.P.); massimiliano.febbi@gmail.com (M.F.); 2 Industrial Engineering Department, University of Tor Vergata, 00133 Rome, Italy; 3 Laboratory of Adapted Motor Activity (LAMA), Department of Public Health, Experimental Medicine and Forensic Science, University of Pavia, 27100 Pavia, Italy; vittoria.carnevalepellino@unipv.it (V.C.P.); caterina.cavallo01@universitadipavia.it (C.C.); alessandro.gatti08@universitadipavia.it (A.G.); matteo.vandoni@unipv.it (M.V.); 4 Department of Physiotherapy, Faculty of Medicine, University of Ostrava, 70103 Ostrava, Czech Republic; 5 Measurement and Movement Laboratory (Me.Mo Lab), NEMOLAB, 20162 Milan, Italy; fabrizio.gervasoni@fisiatramilano.it; 6 National PhD Programme in One Health Approaches to Infectious Diseases and Life Science Research, Department of Public Health, Experimental and Forensic Medicine, University of Pavia, 27100 Pavia, Italy; 7 Minimally Invasive Orthopaedic Surgery Unit, Healthcare Institute “Città di Pavia”, 27100 Pavia, Italy; emanuele.caldarella@grupposandonato.it (E.C.); francescadecaro14@gmail.com (F.d.C.); 8 S.C. Epidemiology, Health Protection Agency of Pavia, 27100 Pavia, Italy; federica.manzoni22@gmail.com; 9 Department of Rehabilitation, Healthcare Institute “Città di Pavia”, 27100 Pavia, Italy

**Keywords:** arthroplasty, indirect neuromuscular electrostimulation, teleprehabilitation

## Abstract

**Background/Objectives**: Hip and knee arthroplasty relieves pain, restores mobility, and improves function in severe joint damage, though pain and strength loss may persist post-surgery. Better pre-surgery function and activity predict improved arthroplasty outcomes. Prehabilitation programs enhance functional abilities, reducing hospitalization duration, and lowering peri-surgery complication risks. This study aims to evaluate the efficacy of four weeks of different modalities of tele-home-prehabilitation programs on perceived pain and functional capacity in patients who are eligible for hip or knee arthroplasty. **Methods**: Forty-four patients (aged 65–80 years) eligible for elective lower limb arthroplasty were enrolled in the present randomized controlled trial study. Participants were randomly assigned to the Electrostimulation Group (EG) or the Home-Based Exercise Group (HG). The EG performed underwent teleprehabilitation program using indirect neuromuscular electrostimulation therapy, while the HG performed home-based exercise supervised by a sports specialist. Functional capacity was assessed with the Timed Up and Go test (TUG), the 30 s Chair Stand test (30CST) and the Six-Minute Walking Test (6MWT). Quality of life was assessed with the Oxford Hip Score (OHS) or Oxford Knee Score (OKS), depending on the participants’ surgery. The Technology Acceptance Model (TAM) questionnaire was completed by the EG after the intervention. **Results**: No significant differences were found among groups in the 30CST and TUG tests. The analyses revealed significant differences for the Oxford Questionnaires and 6MWT. **Conclusions**: Our study highlights the potential of teleprehabilitation using indirect neuromuscular electrostimulation to improve walking autonomy and quality of life of individuals who are candidates for lower limb arthroplasty.

## 1. Introduction

Hip or knee arthroplasty (AP) surgeries have increased in the last few years, accounting for more than 70% of all the AP in Italy in 2019 [1]. Although these surgeries are generally associated with positive outcomes, patients may occasionally experience complications such as pain and loss of strength. These issues can include muscular debilitation (57%), contracture (32%), limb length discrepancy (11%), and malalignment (10%) [2]. Recent studies indicate that higher pre-surgical functional abilities and higher physical activity levels are strong predictors of better prognosis after AP [3]. In particular, adequate levels of joint mobility, lower-limb muscle strength and general functional capacities are critical factors for improved outcomes in older adults undergoing AP surgeries [4]. Recent research in this field has focused on identifying the most effective strategies to minimize post-AP complications. One promising approach is prehabilitation (prehab), an innovative intervention for AP. Prehab consists of a multidisciplinary intervention aimed at improving functional abilities to better manage post-AP complications such as pain, increased days of hospitalization and reduced functional capacity [5,6,7].

Exercise is relevant but prehab interventions typically incorporate nutritional optimization, psychological support, and the management of medical risk factors, as stated in Eras guidelines [8]. All of these components work synergistically to maximize the patient’s preparedness and improve overall outcomes. The specific exercises prescribed often include aerobic training to improve cardiorespiratory fitness, resistance exercises to build muscle strength, and mobility exercises aimed at optimizing joint range of motion [9,10]. These programs are carefully customized to the patient’s baseline physical condition, ensuring both effectiveness and safeness. Studies have shown that even relatively short prehab programs (e.g., 2 weeks) can significantly improve preoperative fitness and reduce postoperative complications [9,10]. However, longer programs (4–6 weeks) consistently demonstrate greater improvements in metrics such as peak oxygen uptake (VO_2_ max), muscle strength, and overall functional capacity. Programs with moderate to high intensity are particularly effective, as they stimulate meaningful physiological adaptations while remaining tolerable for patients. Sessions typically last 20–30 min, 3–5 times per week [9,10].

Many studies support the crucial role of prehab to better handle pain, improve physical function after AP and manage the hospital costs [11,12,13]. Prehab interventions for AP can involve different strategies depending on the primary aim of the prehab. The majority of these include physical exercise to help physical function and weight loss [6,14], balance and gait training interventions to improve the posture in static conditions and walking pattern [15]. Indeed, physical therapy is a valuable adjunct to the treatment of orthopedic conditions [16]. It promotes muscle strength and muscular mass gain, thereby reducing shear forces on joints and mitigating the risk of disuse atrophy [17]. Additionally, regular physical activity provides a wide range of benefits, encompassing enhanced cardiovascular fitness, functional capacity, and quality of life [18]. Other studies support the use of electrostimulation, cryotherapy, pressure therapy and passive mobilization to achieve a better prognosis [19]. This approach not only improves the functional status and quality of life of patients but also contributes to reducing health system costs [20]. Indirect neuromuscular electrostimulation (NEMS) has demonstrated efficacy in the clinical management of orthopedic conditions by facilitating the restoration of muscle strength [21].

Both modalities are extensively used in prehab programs to enhance muscle strength and functional capacity, yet they differ significantly in their mechanisms of action [22]. While traditional exercise improves muscle function through active participation and progressive overload, NEMS is beneficial for patients with limited mobility or significant muscular weakness, as it avoids voluntary activation and directly stimulates muscle contraction [10,22]. While other forms of physiotherapy, such as cryotherapy and passive mobilization, are often incorporated into prehab programs, they primarily address only specific aspects of recovery like pain management and joint mobility [9].

NEMS has shown substantial benefits in prehab settings, resulting in significant improvements in muscle function and shortened postoperative recovery time, particularly following knee AP [15,16]. Previous studies have demonstrated that both clinical electro-stimulators and portable devices are equally effective in improving muscle strength [23,24]. However, prehab programs face several limitations, including accessibility to treatment facilities, sustaining patient motivation, standardizing procedures, and managing pain [4,12] leading to reduced adherence to prehab programs with reduced benefits. Nowadays, teleprehabilitation (teleprehab) has emerged as a powerful alternative to traditional face-to-face programs [24]. This intervention has already been integrated into prehab programs to enhance patients’ functional capacities and quality of life, while also reducing healthcare system costs [25]. Teleprehab programs have shown positive effects, including increased strength and improved condition of muscles, tendons, and ligaments, which help attenuate the shear forces at joint level. In addition, these programs have demonstrated a preventive effect against hypotrophy caused by reduced mobility, while enhancing cardiovascular fitness, functional status, quality of life and reducing pain [17]. For these reasons, the aim of this study was to evaluate the efficacy of four weeks of two different modalities of teleprehab programs on perceived pain and functional capacity in patients who are eligible for hip or knee AP.

## 2. Materials and Methods

### 2.1. Study Design and Participants

A randomized controlled trial was conducted between April and August 2024. Forty-four patients eligible for elective lower limb AP were enrolled from the Minimally Invasive Surgery Unit of the Department of Orthopedic Surgery at the Healthcare Institute “Città di Pavia” (HICP), Pavia, Italy. The study protocol was approved by the Lombardy 6 Territorial Ethics Committee on 24 January 2024 (Prot. 0003762) and registered on ClinicalTrials.gov on 13 April 2024 (NCT06363643). All the procedures were conducted in accordance with the Declaration of Helsinki (1975, revised 2013) [26].

Inclusion criteria were age between 65 and 80 years and undergoing elective knee or total hip AP with a posterolateral approach; ability to use technology; ability to clearly understand equipment, instructions, and ability to read and understand the informed consent forms. All the participants provided written and verbal informed consent after receiving detailed study information. The participants were informed that they could withdraw from the study at any moment without any consequences. Participants were randomly assigned, by cluster randomization, to two different groups: the Electrostimulation Group (EG) and the Home-Based Exercise Group (HG). To ensure that the comparison groups were of the same size, a block randomization with a variable-size random sequence prepared by the Medical Biostatistician contact person was employed. For the creation of the block design, random number generation was performed using STATA statistical software (Release 17.0, 2021, Stata Corporation, College Station, TX, USA) with the ’ralloc’ command. The EG followed a teleprehab program incorporating indirect electrostimulation, supervised by an operator, while the HG engaged in home-based exercises supervised by a sports specialist. Both groups underwent a four-week teleprehab program consisting of 30 min therapy sessions conducted three times per week via the Google Meet online platform (Google LLC, Mountain View, CA, USA). Before the start of the study, all participants received clear instructions on how to use the electrostimulation device and how to perform the exercises with the correct technique. Before the start of the study protocol (T0), the functional capacity of the participants was evaluated. The assessments were also performed at the end of the 4-week programs (T1), before undergoing AP. Four participants withdrew from the study due to a change in surgery date.

### 2.2. Study Protocol

**Figure 1:**
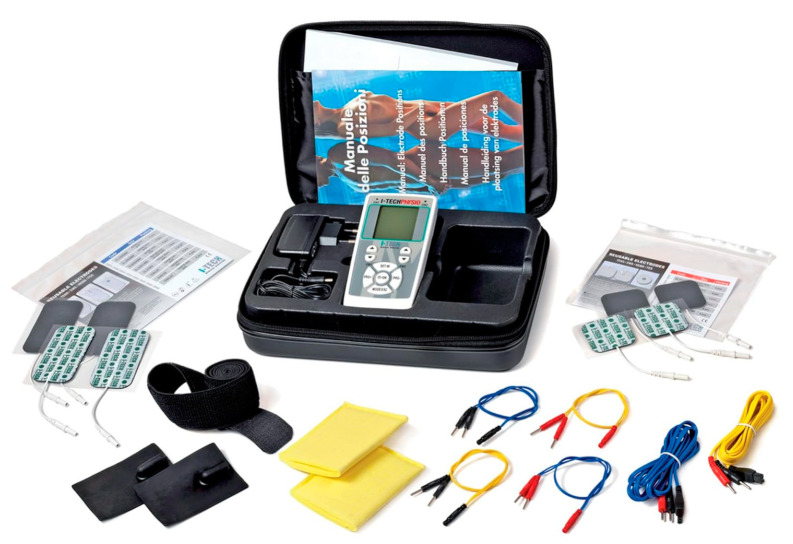
Medical device electrostimulation.

The EG performed teleprehab using NEMS therapy supervised by a physiotherapist. Participants employed a programmable medical device (I-TECH PHYSIO, IACER srl, Scorzè, Venice, Italy) (Figure 1) equipped with two independent channels and four pairs of pre-gelled 130 × 76 mm electrodes (TOP-RANK Health Care Co., Ltd., Shaoxing, China). The device was delivered directly to the patients’ home and picked up at the end of the program.

**Figure 2:**
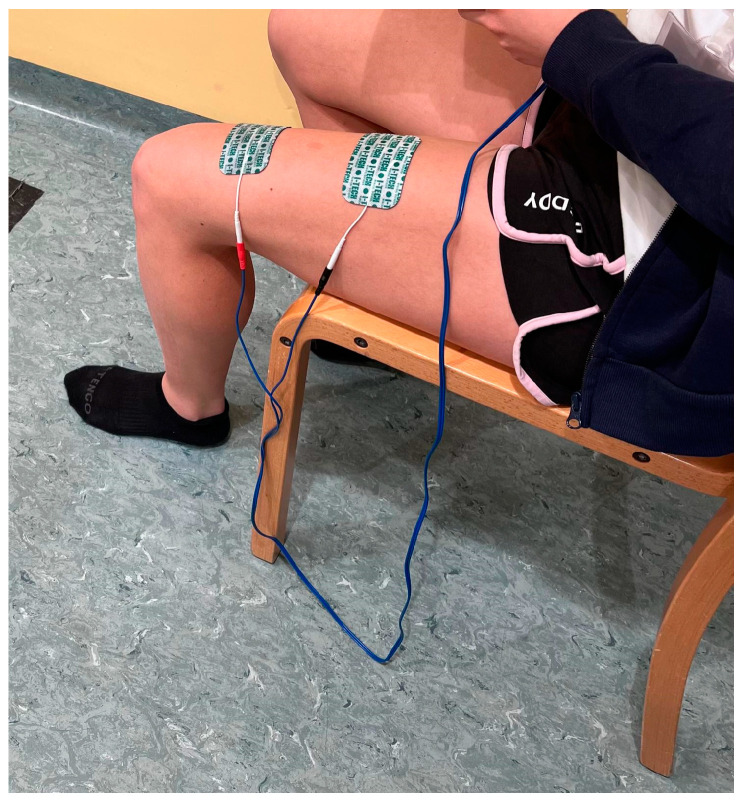
Electrode position.

During the supervised session, a single channel was utilized with electrodes positioned on the quadriceps (Figure 2): the lower electrode 4 cm above the patella and the upper electrode 4 cm above the lower one.

Patients were restricted to a single 45 Hz program with pre-set parameters (Table 1) and received detailed safety instructions from the physiotherapist. Stimulation intensity was progressively increased throughout the sessions. The total treatment time was 30 min. The muscle contractions lasted 6 s with a 10 s rest and a pulse width of 300 µs.

The HG performed 30 min of home-based exercise three times per week supervised by a sports specialist using an online platform. The training session consisted of a total body warm-up with mobility exercises, bodyweight resistance training with 6 exercises of 3 sets for 10–12 repetitions for the major muscle groups of the lower body and a final cool-down with respiratory exercises. The training program maintained the same intensity and resistance levels during the entire intervention and consisted of the following exercises: leg extension while seated, lower limb extension while standing, closed box squat, plank to the wall, hip abduction and calf raise.

### 2.3. Physical Assessment

**Figure 3:**
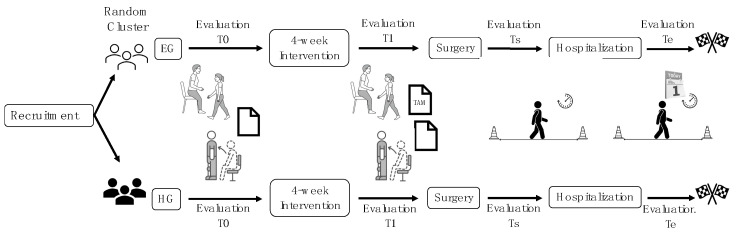
Timeline of physical assessment. Legend: TUG, Timed Up and Go test (seconds, s); 30CST, 30 s Chair Stand test (number of repetitions, n); OKS, Oxford Knee Score; OHS, Oxford Hip Score (0 to 48 points); TAM, Technology Acceptance Model (0 to 28 points); 6MWT, Six-Minute Walking Test (meters, m). T0 = Before the start of the study protocol; T1 = at the end of the 4-week program; Ts = admission to the rehabilitation ward and Te = discharge.

To evaluate the effects of the two programs, a battery of tests and questionnaires was administered online at T0 and T1. To assess the functional capacity, the Timed Up and Go (TUG) test and 30 s Chair Stand test (30CST) were performed. The quality of life was assessed with the Oxford Hip Score (OHS) or Oxford Knee Score (OKS), in accordance with participants’ surgeries. The Six-Minute Walking Test (6 MWT) was conducted on admission to the rehabilitation ward (Ts) and at discharge (Te) to evaluate walking autonomy. The Technology Acceptance Model (TAM) questionnaire was completed by the experimental group (EG) at T1 to assess technology usability, pleasantness, and attitude to reuse. Additionally, total hospitalization days were recorded at Te. The timeline of the physical assessment is shown in Figure 3.

#### 2.3.1. Timed Up and Go (TUG) Test

The TUG test is a simple, reliable, and cost-effective way to assess overall functional mobility. To perform the test, patients started seated with their back against the chair. They were instructed to walk at their normal pace. The test involved standing up from the chair, walking 3 m, turning around, returning to the chair, and sitting back down. The time taken to complete the task was recorded in seconds [27].

#### 2.3.2. 30 s Chair Stand Test (30CST)

The 30CST test involves recording the number of sit-to-stand repetitions performed in 30 s. Subjects were in the sitting position with arms crossed over the chest. After the cue, the patients started to perform the repetitions as quickly as possible from the sitting position touching the chair to the full standing position. The total number of completed sit-to-stand repetitions during the 30 s period was recorded [28].

#### 2.3.3. Oxford Hip Score (OHS) or Oxford Knee Score (OKS)

These questionnaires were used to assess pain and functioning in patients undergoing lower limb AP [29]. OHS consists of 12 items assessing pain, mobility, activities of daily living, and emotional well-being, while the OKS focuses on pain, stiffness, physical function, and social function. Each item is rated on a 5-point scale (0 to 4), with higher scores indicating better function. The total score ranges from 0 to 48, with higher scores reflecting better hip or knee function. In clinical practice, both the OHS and OKS are used to monitor patient outcomes before and after surgery, providing objective data that inform clinical decision-making and offer insights into recovery and quality of life. Scores are interpreted as follows: 0–24 indicates severe disability, 25–40 moderate disability, and 41–48 mild disability or near-normal function. These tools are essential for assessing the effectiveness of orthopedic treatments and improving patient care [30].

#### 2.3.4. Technology Acceptance Model (TAM)

The TAM assesses usability and perceived pleasure while using the technology. It defines the degree to which a person believes that using a certain technology will improve their daily routine, so if a technology is perceived as useful, the user is likely to use it. It consists of 22 items, divided into 4 domains. The scores range from 1 to 7, where 1 corresponds to “totally dissatisfied” and 7 corresponds to “totally satisfied”. The total score is 28. Patients had to read the questions carefully [31].

#### 2.3.5. Six-Minute Walking Test (6MWT)

The 6MWT was performed according to the guidelines published by the American Thoracic Society [32]. Patients were asked to walk 30 m along a corridor marked with tape. They were instructed to walk for six minutes at a comfortable pace, avoiding running, and to keep moving for the entire duration if possible. Evaluators provided a time update at the three-minute mark and instructed the patients to stop as soon as the six minutes ended. The total distance covered was measured in meters and recorded on a spreadsheet for further analysis. Patients were allowed to rest during the test trial if necessary, but the time was not stopped [33].

### 2.4. Statistical Analysis

A total number of 40 patients (20 per group) allows us to identify an approximate 90% success rate in the EG versus a 50% rate in the HG. Success is defined as an improvement of at least 11% at the 30CST. The sample size was calculated by Pearson’s chi-square test for two independent proportions, considering an alpha error of 0.05, a power of 80% and an effect size of 1.76 (according to Cohen’s classification). Considering a dropout rate of 10%, a total number of 44 patients will need to be recruited (22 per group).

Data were expressed as mean (95% confidence interval, CI). A generalized linear mixed for repeated measures was applied to analyze intervention effects on primary and secondary endpoints. This approach was selected to adjust both parametric and non-parametric data, using a Gaussian or a Gamma distribution depending on the data nature. Intervention effects were assessed through a generalized linear mixed-effects model, using individual growth measures as a function of the randomization effect, age, sex and the interaction between group and time.

For all analyses, a *p*-value < 0.05 was considered statistically significant. The beta coefficient was presented as both non-standardized and standardized. Analyses were conducted using R software (version 4.4, R Foundation for Statistical Computing). The “glmer” package was utilized for the generalized linear mixed model, the “Durga” package for group comparison plots, and the “emmeans” package for post hoc parametric comparisons.

## 3. Results

Descriptive characteristics and within-group comparisons are presented in Table 2.

Statistical analyses did not reveal differences among groups in the 30CST and TUG tests with a mean difference (EG vs. HG) of 0.10 (95% CI: −1.10, 1.30; *p* = 0.867; effect sizes (ES): 0.196) and −0.31 (95% CI: −1.37, 0.76; *p* = 0.562; ES: 0.211), respectively.

The Oxford Questionnaires and 6MWT improved more in the EG compared to the HG with a mean difference (EG vs. HG) of 4.20 (95% CI: 1.17, 7.23; *p* = 0.008; ES: 0.142) and 41.90 (95% CI: 3.95, 79.84; *p* = 0.030; ES: 0.405), respectively.

Results derived from the statistical comparison of the performances of the two groups are presented in Table 3.

**Figure 4:**
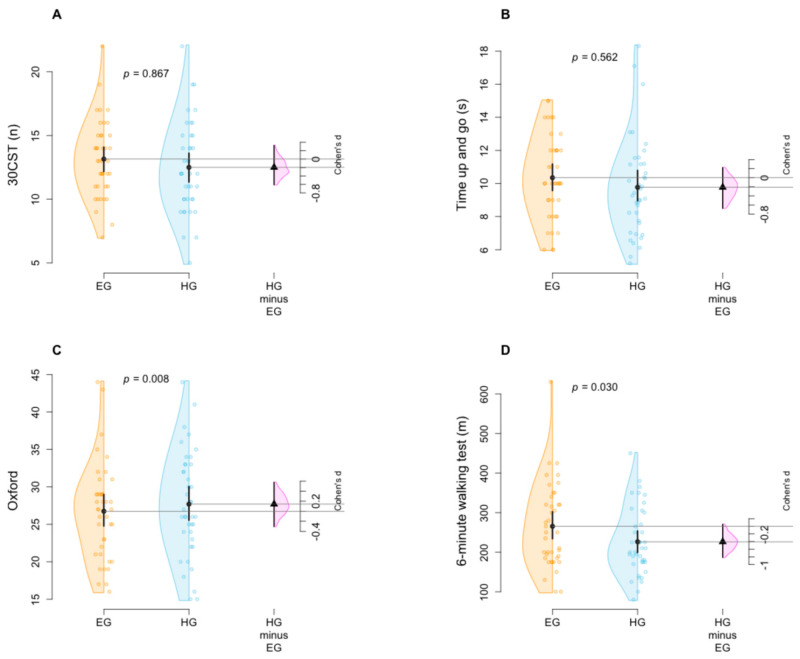
The 30 s Chair Stand test (30CST, (**A**)), Time Up and Go (**B**), Oxford scale (**C**) and Six-Minute Walking Test (**D**) differences are displayed by Gardner–Altman plots, which show a representation of observed values by the two-group comparison (EG vs. HG), a bootstrap effect size (Cohen’s d) estimation, mean and 95% confidence interval. Two-sided *p* values were obtained from a generalized linear mixed model evaluating the differences between groups adjusting for sex and age.

For further clarity, a graphical representation of the comparison of the results can be found in Figure 4.

## 4. Discussion

This study aimed to evaluate the efficacy of four weeks of teleprehab with NEMS on perceived pain and functional capacity in patients eligible for AP.

Both the EG and HG showed improved performances in their 6MWT scores, exceeding the minimal clinically significant difference of a 30 m increase with 50% and 73% increases, respectively [34]. Also, the Oxford Questionnaire showed better outcomes for the EG compared to the HG. Our findings align with previous studies that have examined the effectiveness of NEMS in prehab patients on aerobic capacity. The scientific literature reported that NEMS allows passive muscle contraction and activity promoting muscle strength gains and endurance [35]. These effects are undoubtedly beneficial for patients who are suffering from muscle weakness or chronic pain, which are common conditions in hip or knee AP candidates [20,36].

Despite sessions lasting only 20 min with a fixed muscle stimulation frequency of 50 Hz [35], Şavkin R. et al. [35] found significant improvements in walking ability among patients undergoing a three-week prehab program utilizing NEMS. Also, a study conducted by Walls RJ et al. confirmed the benefits of thrice-weekly 45 min sessions of NEMS on functional recovery after AP surgery [20]. In contrast to our findings, Savikin et al. reported an improvement in walking capacity only after a longer duration of the NEMS protocol (12 weeks). This discrepancy could be attributed to the shorter duration of their sessions with NEMS that lasted only 20 min compared to 30 min performed in our protocol [35]. Moreover, our program differed in key stimulation parameters. While Şavkin et al. [35] used a fixed frequency of 50 Hz and Walls RJ et al. [20] followed protocols with longer contraction periods and higher session durations, our protocol employed a frequency of 45 Hz with a pulse duration of 300 μs and a 6 s contraction/10 s rest cycle. These parameters may have optimized muscle activation and contributed to the equivalent or superior results observed over a shorter timeframe in our study, highlighting the potential benefits of fine-tuning NEMS settings for functional recovery.

Although no statistically significant differences were found between groups, HG and EG demonstrated improvements in the performance of the TUG and 30CST tests after the end of the program. Similarly to our findings, Saviski et al. did not observe differences in the 30CST either at the fourth or sixth week between the two groups [35]. These findings suggest that both interventions contributed to enhanced functional performance, highlighting the potential benefit of the teleprehab protocol to improve mobility and strength. Our findings demonstrated that both treatments were successful, and all participants completed more than 11 or 12 sessions. The level of adherence could suggest that NEMS interventions were not only well-tolerated but also highly accepted by participants, contributing to positive outcomes after the protocol. This might describe a certain degree of willingness to use and/or reuse the technology, as already found in similar studies [36]. Indeed, in our study, patients reported high levels of satisfaction with the system’s accessibility and usability, as measured by the TAM. The positive perception reported by participants is consistent with findings from previous studies, suggesting that patients are willing to adopt this technology due to its ease of use and effectiveness in minimizing pain levels [37]. Indeed, several studies support the use of NEMS, which not only optimizes resource utilization, but also effectively manages the rising number of patients seeking prehab [38]. This innovative approach is patient-centered, supporting specifically those people who face logistical barriers or are particularly fragile, and it aims to provide timely and personalized prehab services [39]. While traditional prehab methods often result in a decline in motivation and effectiveness, the NEMS-based teleprehab approach seems to be a promising solution to overcome this issue [40]. The Oxford Questionnaire, as confirmed by Hamilton et al. [29], has proven to be a valuable tool for evaluating the quality of life and in the pain relief after AP surgery. Our study found improvements in these outcomes for patients in both groups, with significantly greater positive results observed in the EG. The patients reported a moderate recovery and a “near-normal” quality of life. Although some patients experienced mild pain or mobility limitations, overall, their quality of life improved substantially compared to what was found before the teleprehab intervention.

We are conscious that this study has some limitations. First of all, the total duration of the protocol might have been a limitation. Indeed, a longer period and having included other muscle groups could have led to additional findings and to potential differences between groups. Secondly, a third group that performs in-person prehab could improve our findings. Finally, we considered only one NEMS program, while a comparison between different therapies could improve the level of understanding of the beneficial effects of this technology.

Despite these limitations, this study highlights significant strengths. The teleprehab approach has the potential to reach a larger number of participants by eliminating barriers such as long travel distances to hospitals, extended therapy durations, and the need for a high number of therapists. The enhanced accessibility and adherence to the program also contribute to significant cost reductions. Additionally, specific work on the lower limbs, including the quadriceps, plays a crucial role in ambulation and overall functional abilities, with significant systemic effects. This muscle group, regularly stimulated in clinical practice after knee arthroplasty, is easily accessible for autonomous electrode application, making it highly suitable for tele-rehabilitation protocols [20,35]. These factors together enhance the feasibility and effectiveness of teleprehab in post-surgical functional recovery. To build on these findings, future research could include different NEMS programs in various populations to provide a more comprehensive understanding of the intervention’s impact in the orthopedic setting.

## 5. Conclusions

The present research highlights the potential of teleprehab using NEMS to improve the care pathway of individuals eligible for AP surgery. While the study’s limitations require careful consideration, the findings suggest that this approach is feasible and may offer several benefits for orthopedics patients. By empowering patients to engage in independent prehab at home, this approach has the potential to optimize preoperative care and enhance overall patient experience. Moreover, Teleprehab has the potential to significantly reduce the costs associated with AP prehabilitation while maintaining accessibility and effectiveness.

## Figures and Tables

**Table 1:** Program pre-set parameters.

Parameter	Time
Total time of treatment	30 min
Pulse width	300 µs
Contraction	6 s
Rest	10 s
Ramp	2 s

Notes: min = minutes, µs = microseconds; s = seconds.

**Table 2:** Descriptive characteristics and within comparison of the EG and HG, respectively.

	EG (n = 20, 12F)		HG (n = 20, 8F)	
	Pre	Post	Pre	Post
Age	67.65 (65.07, 70.23)	-	68.15 (65.54, 70.76)	-
Number of sessions (n)	-	11 (11, 12)	-	12 (12, 12)
Hospitalization days (n)	14.20 (12.42, 15.98)	-	14.20 (13.01, 15.39)	-
30CST (n)	11.95 (10.78, 13.13)	14.35 (13.07, 15.63) *	11.35 (9.80, 12.90)	13.65 (12.19, 15.11)
TUG (s)	11.40 (10.30, 12.51)	9.30 (8.38, 10.22) *	10.67 (9.18, 12.15)	8.87 (7.93, 9.82) *
Oxford scale	23.75 (21.13, 26.38)	29.75 (27.33, 32.17) *	26.80 (23.525, 30.08)	28.60 (25.78, 31.42) *
6MWT (m)	194.25 (170.44, 218.06)	336.50 (292.89, 380.11) *	180.75 (153.46, 208.04)	271.75 (236.39, 307.12) *
TAM	-	26.26 (25.69, 26.84)	-	-

Unless otherwise stated, data are presented as mean (95% confidence interval, CI). * pre-post *p*-value < 0.05. 30CST = 30 s Chair Stand test, TUG = Time Up and Go, 6MWT = Six-Minute Walking Test; TAM = Technology Acceptance Model.

**Table 3:** The EG and the supervised training groups differ in physical test performance.

	Difference Between Groups (EG–HG)		
	Mean Difference (95% CI)	*p*-Value	Cohen’s d
30CST (n)	0.10 (−1.10, 1.30)	0.867	0.196
TUG (s)	−0.31 (−1.37, 0.76)	0.562	0.211
Oxford scale	4.20 (1.17, 7.23)	0.008 *	0.142
6MWT (m)	41.90 (3.95, 79.84)	0.030 *	0.405

Data are presented as mean differences with 95% CI between groups, calculated by computing the difference between EG vs. HG (i.e., EG minus HG). Two-sided *p* values were obtained from a generalized linear mixed model evaluating the differences between groups adjusting for sex and age. * *p*-value < 0.05. 30CST = 30 s Chair Stand test, TUG = Time Up and Go, 6MWT = Six-Minute Walking Test.

## Data Availability

All data can be obtained from the corresponding author upon reasonable request.
